# Apical and basal epitheliomuscular F-actin dynamics during *Hydra* bud evagination

**DOI:** 10.1242/bio.022723

**Published:** 2017-06-19

**Authors:** Roland Aufschnaiter, Roland Wedlich-Söldner, Xiaoming Zhang, Bert Hobmayer

**Affiliations:** 1 Department for Evolutionary Developmental Biology, Institute of Zoology and Centre for Molecular Biosciences, University of Innsbruck, Technikerstr. 25, A-6020 Innsbruck, Austria; 2 Max-Planck-Institute of Biochemistry, Research Group Cellular Dynamics and Cell Patterning, Am Klopferspitz 18, D-82152 Planegg, Martinsried, Germany; 3 Department of Anatomy and Cell Biology, University of Kansas Medical Centre, Kansas City, KS 66160, USA

**Keywords:** Lifeact, Epithelial cell, Morphogenesis, Cnidarian, Tissue evagination, Evolution

## Abstract

Bending of 2D cell sheets is a fundamental morphogenetic mechanism during animal development and reproduction. A critical player driving cell shape during tissue bending is the actin cytoskeleton. Much of our current knowledge about actin dynamics in whole organisms stems from studies of embryonic development in bilaterian model organisms. Here, we have analyzed actin-based processes during asexual bud evagination in the simple metazoan *Hydra*. We created transgenic *Hydra* strains stably expressing the actin marker Lifeact-GFP in either ectodermal or endodermal epitheliomuscular cells. We then combined live imaging with conventional phalloidin staining to directly follow actin reorganization. Bending of the *Hydra* epithelial double layer is initiated by a group of epitheliomuscular cells in the endodermal layer. These cells shorten their apical-basal axis and arrange their basal muscle processes in a circular configuration. We propose that this rearrangement generates the initial forces to bend the endoderm towards the ectoderm. Convergent tissue movement in both epithelial layers towards the centre of evagination then leads to elongation and extension of the bud along its new body axis. Tissue movement into the bud is associated with lateral intercalation of epithelial cells, remodelling of apical septate junctions, and rearrangement of basal muscle processes. The work presented here extends the analysis of morphogenetic mechanisms beyond embryonic tissues of model bilaterians.

## Abstract (precis)

**Summary:** Transgenic Lifeact-GFP *Hydra* polyps provide a powerful system for live imaging of actin dynamics during morphogenesis and development of a simple and ancestral animal model.

## INTRODUCTION

Bending and folding of epithelial tissues are crucial determinants in creating animal shape, often initiate asexual reproduction, and represent fundamental morphogenetic mechanisms underlying the formation of multiple inner organs such as the gut, the neural tube and extremities (Gierer, 1977). The physical forces that lead to shape changes in tissues are created by the coordinated activities of individual cells including cell shape changes, cell motility, asymmetric cell division or changes in cell adhesion (Trinkaus, 1969). At the molecular level, dynamic cell behaviour is largely based on the actin cytoskeleton either via directed polymerization of actin filaments or through interaction of actin filaments with various binding partners such as myosin motors (Heisenberg and Bellaiche, 2013; Guillot and Lecuit, 2013; Munjal and Lecuit, 2014). Our understanding of dynamic actin processes is most advanced *in vitro* in cultured cells and in tissues of developing bilaterians. Improved imaging techniques and the successful use of fluorescent markers for actin and actin-related proteins have proven to be powerful tools to dissect the cellular and biomechanical basis of morphogenesis in model organisms such as *Drosophila*, sea urchin, zebra fish and *Xenopus* (Edwards et al., 1997; Franke et al., 2005; Burkel et al., 2007; Skoglund et al., 2008; Solon et al., 2009; Martin et al., 2009; Martin and Goldstein, 2014; Lukinavičius et al., 2014). Lifeact, a 17-amino acid actin-binding peptide from budding yeast, is a particularly promising actin-binding probe (Riedl et al., 2008). It appears not to interfere with F-actin dynamics in cellular processes, lacks competition with endogenous actin binding proteins, and permits analysis of *in vivo* F-actin dynamics (Riedl et al., 2008; Spracklen et al., 2014; Lemieux et al., 2014; DuBuc et al., 2014). Lifeact binds to actin filaments with high specificity in yeast, filamentous fungi, plants and various metazoans. In the current study, we report the generation of stable transgenic *Hydra* expressing Lifeact-GFP and use these animals to study tissue evagination during asexual reproduction in a simple model system.

*Hydra* is a member of the diploblastic phylum Cnidaria, the sister group to the bilaterian clade. The *Hydra* polyp exhibits one major oral-aboral body axis and its body wall*,* akin to that of other cnidarians, is formed by two epithelial layers separated by extracellular matrix (‘mesoglea’). The individual unit of each tissue layer is an epitheliomuscular cell, which is commonly called an epithelial cell in the *Hydra* literature. Different from developing epithelia in higher bilaterians, *Hydra* epithelial cells possess muscle processes located directly adjacent to the mesoglea (Mueller, 1950). Within each layer, epithelial cells are connected to their neighbours apically via belt-like septate junctions and basally at the level of the muscle processes via multiple desmosome-like junctions. Furthermore, the basal ectodermal cell membrane is connected to the mesoglea via hemidesmosome-like junctions. Ectodermal muscle processes run along the primary oral-aboral (mouth-foot) axis of the animal, endodermal muscle processes run perpendicular to this axis. Based on their patterns of connection and their differential orientation in the two layers, muscle processes control contraction-elongation behaviour, feeding and peristaltic gut movements. A third cell line, the interstitial stem cell system, gives rise to nerve cells, gland cells, nematocytes and germ cells, but cells of this lineage are not directly involved in shaping, maintaining, or regenerating the animal's body wall (Marcum and Campbell, 1978; Sugiyama and Fujisawa, 1978).

Asexual bud formation is the primary mode of reproduction in *Hydra*. The body wall of an adult polyp evaginates in the lower gastric region, and the resulting bud anlage elongates perpendicular to the mother polyp's body axis and eventually forms a complete small animal that finally detaches. Throughout the entire budding process, both epithelial layers keep their unicellular conformation intact. Bud formation starts in a circular, thickened area in the lower gastric region of the mother polyp (Otto and Campbell, 1977). Histological analysis revealed that a group of endodermal epithelial cells in the centre of the thickened area undergoes pronounced apical-basal shortening and starts to protrude into the ectodermal layer (von Gelei, 1925; Philipp et al., 2009). Thereafter, both tissue layers evaginate. Coordinated movement of epithelial cells from all directions towards the evaginating centre is responsible for transforming an initially flat sheet of parental body wall into the tubular shape of an evaginating bud. This recruitment of parental tissue occurs up to bud stages 6-7 (Otto and Campbell, 1977). Up to this phase, enhanced cell proliferation and oriented cell division have been shown to play no role in shaping the bud (Clarkson and Wolpert, 1967; Webster and Hamilton, 1972; Otto and Campbell, 1977; Holstein et al., 1991; Shimizu et al., 1995). Only in later budding stages does tissue growth contribute to maturation of the bud (Otto and Campbell, 1977). Ectodermal epithelial cells have been shown to intercalate laterally during budding (Philipp et al., 2009), similar to convergent extension movements of mesenchymal cells in *Xenopus* or germ band elongation in *Drosophila* (Keller et al., 2000; Rauzi et al., 2008). It has been speculated that basal muscle processes act in epithelial cell motility and bud morphogenesis, but the histological methods used to stain actin or other cytoskeletal elements offered only limited resolution and did not allow live imaging (Otto, 1977; Campbell, 1980).

Although *Hydra* bud formation has been intensively studied in the past, core issues remain unanswered. The behaviour of single epithelial cells during tissue evagination is not well understood. Furthermore, the contribution of each of the two epithelial layers to the budding process and the detailed dynamics of actin structures as operators of the developing 3D structure are unresolved. In order to approach these topics, we aimed at developing a way to visualize the actin cytoskeleton *in vivo* in *Hydra* epithelial cells, to track dynamic changes of actin networks during tissue bending and movement into evaginating buds, and to deduce from these observations possible underlying forces that drive morphogenesis. We created two transgenic *Hydra* strains, in which Lifeact-GFP localized specifically to actin filaments in ectodermal and endodermal epithelial cells, revealing previously undescribed F-actin structures. *In vivo* tracking of Lifeact-GFP combined with phalloidin staining on fixed samples revealed distinct patterns of reorientation of muscle processes, remodelling of actin-networks at apical septate junctions, and the formation of muscle process-like structures associated with motility. These newly generated transgenic animal strains have the potential to provide an understanding of cytoskeletal dynamics not only during bud morphogenesis, but also during the establishment of epithelial polarity and regeneration.

## RESULTS

### Transgenic *Hydra* expressing Lifeact-GFP

The *Hydra* genome encodes several actin and actin-like genes (XM_002154426, XM_002154660, XM_012700059, XM_002158909, XM_002158614/Sc4wPfr_228.g20688, XM_002160174/ Sc4wPfr_422.g20281). The two most conserved actin genes (named here actin1: XP_002154462, Fisher and Bode, 1989; actin2: XP_002154696) are different to each other in their predicted protein sequence at only three amino acid positions, but are substantially different at the nucleotide level. Both genes are uniformly expressed throughout the body column with higher mRNA levels in the ectodermal layer (Fig. S1).

**Fig. 1.**
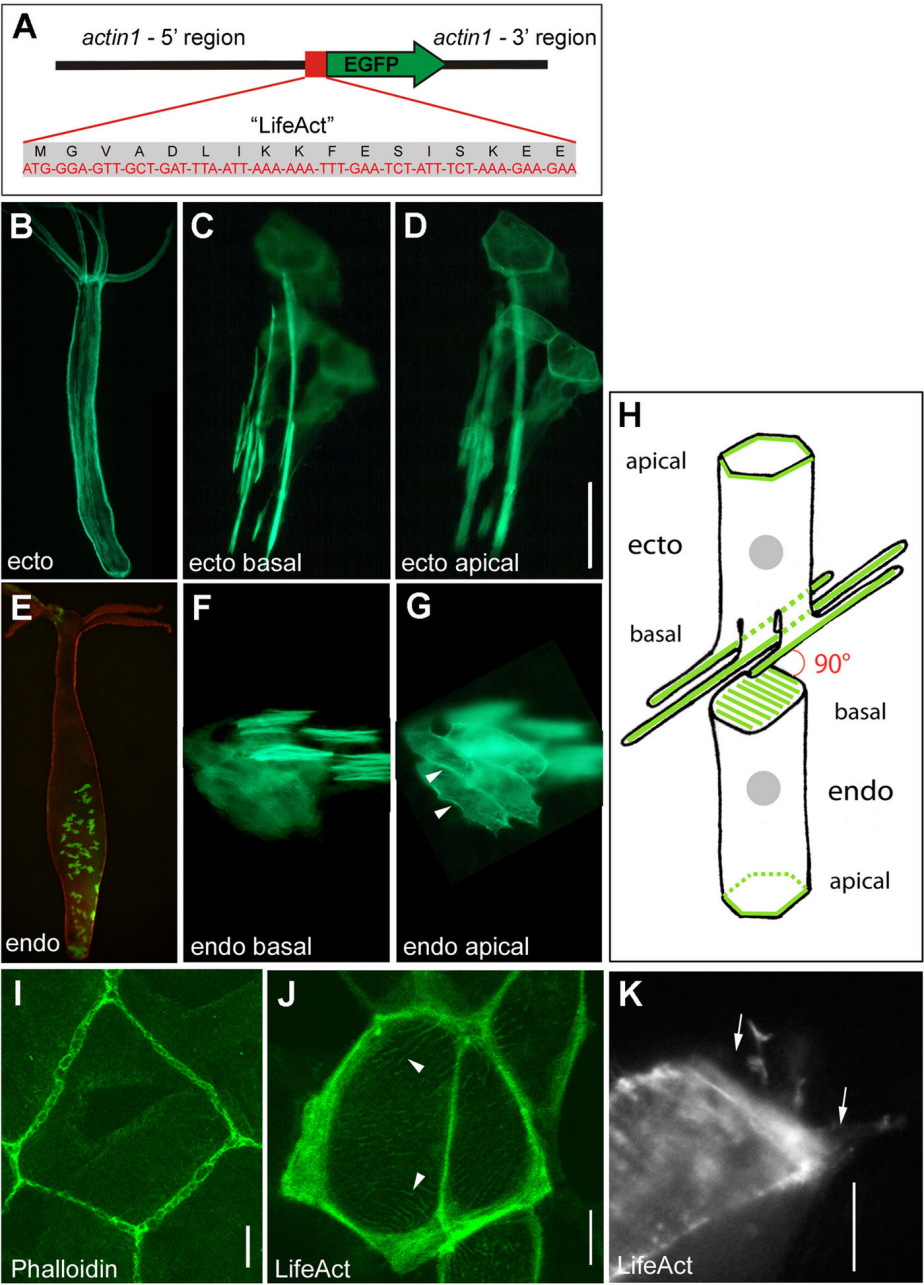
**Transgenic *Hydra* strains expressing Lifeact-GFP.** (A) Codon-optimized sequence of the *lifeact-GFP* expression construct driven by the *Hydra actin1* promoter. (B) *Hydra* polyp with mosaic expression of Lifeact-GFP in large areas of the ectodermal layer. (C) Cluster of three transgenic ectodermal epithelial cells with focus level at the basal muscle processes running along the primary oral-aboral axis; (D) the same cell cluster with focus at apical cell junctions. (E) *Hydra* polyp with mosaic expression of Lifeact-GFP in the endodermal layer. (F) Cluster of four transgenic endodermal epithelial cells with focus level on basal muscle processes; (G) the same cell cluster with focus at apical cell junctions. (H) Schematic drawing of an ectodermal and an endodermal epithelial cell as positioned in the body wall of *Hydra* with prominent actin structures highlighted in green. Basal muscle processes in ectodermal and endodermal epithelial cells are oriented perpendicular to each other. The mesoglea linking both tissue layers is not included. The connections between ectodermal and endodermal epithelial cells across the mesoglea are also not shown. (I-K) F-actin structures at the apical surface of ectodermal epithelial cells. (I) Confocal projection (3.5 µm depth) at apical cell junctions in fixed tissue stained with Alexa Fluor 488 Phalloidin. (J) Corresponding confocal section at apical cell junctions in a living mosaic Lifeact-GFP polyp. (K) Actin-based apicolateral lamellipodia-like structures (arrows) as visualized in a single frame of a movie taken with a TIRF microscope in a living mosaic Lifeact-GFP polyp (see Movie 2). The black space around the transgenic cell is occupied by nontransgenic epithelial cells. Scale bars: 50 µm in C, D, F and G; 10 µm in I-K.

In order to visualize the actin cytoskeleton *in vivo*, we generated transgenic polyps expressing Lifeact-GFP under the control of the *Hydra actin1* promoter (Fig. 1A). Expression constructs were injected into embryos at the one- to two-cell stage. Later, hatching polyps strongly expressed Lifeact-GFP in patches of ectodermal or endodermal epithelial cells derived from embryonic precursor cells that had by chance incorporated the construct into their genomic DNA. These mosaic Lifeact-GFP polyps were selected and propagated asexually (Fig. 1B,E). They exhibited normal morphology and behaviour, and were capable of asexual and sexual reproduction. They produced buds with the same frequency as wild-type animals. Hence, Lifeact-GFP expression did not seem to critically interfere with major physiological and developmental processes (see Discussion). To detect whether Lifeact-GFP fusion proteins specifically localized to the actin cytoskeleton, transgenic animals were fixed and counterstained with rhodamine-phalloidin. In these preparations, we found complete overlap of the two labels at the site of actin-based structures (Fig. S2).

### Architecture of the actin cytoskeleton in *Hydra* epithelial cells

Mosaic animals with only few Lifeact-GFP positive cells proved to be particularly well suited for observations of single cells in intact tissue. In these polyps, the F-actin cytoskeleton could be visualized at unprecedented resolution. As observed earlier, muscle processes at the base of epithelial cells represented the most prominent actin structures. In the ectoderm, they were organized in discrete, condensed fibres running along the oral-aboral axis of the polyp (Fig. 1C,H). Their length was variable (Fig. 1C) and ranged between one and five times the planar epithelial cell diameter as measured at the apical surface. One ectodermal epithelial cell possessed on average two to three basal muscle processes (2.4±0.6, *n*=52 cells) (Fig. 1C), consistent with earlier observations made by electron microscopy (Mueller, 1950).

In the apical part of ectodermal epithelial cells, Lifeact-GFP was concentrated in a circumferential belt of actin filaments at the position of septate junctions (Fig. 1D,H). In fixed, phalloidin-labelled specimens, this structure resembled a chain-like arrangement (Fig. 1I). In living Lifeact-GFP cells (Fig. 1J), however, it appeared as a continuous belt indicating that the chain-like arrangement is an artefact caused by chemical fixation. In addition, a fine network of cortical actin filaments stretched across the width of the cell under the apical membrane (Fig. 1J, arrowheads). High resolution live imaging using total internal reflection fluorescence (TIRF) microscopy revealed continuous movement within this cortical actin network (Movie 1). TIRF microscopy also revealed filopodia-like protrusions extending laterally at the most apical region (arrows in Fig. 1K; Movie 2). These protrusions appear to represent the cytoplasmic extensions frequently found in transmission electron microscopic images connecting neighbouring cells via septate junctions (Fig. S3).

Muscle processes at the basal face of endodermal epithelial cells were oriented perpendicular to the oral-aboral axis of the polyp. They occurred in much larger numbers (commonly 10 or more in a relaxed cell), covered the entire basal surface, and did not extend significantly beyond the periphery of the cell (Fig. 1F,H). In phalloidin-stained preparations, endodermal muscle processes were obscured by the much brighter ectodermal fibres (Fig. S2). As in the ectoderm, we found Lifeact-GFP-labelled actin rings at the sites of endodermal septate junctions (Fig. 1G,H). However, it was not possible to study the apical endodermal region using confocal or TIRF microscopy due to the thickness of the epithelial double layer.

### Initiation of bud formation involves reorientation of endodermal muscle processes

The oral-aboral axis of the developing bud is formed at a right angle to the axis of the parental polyp. As a consequence, muscle processes in both the ectoderm and endoderm have to reorganize in order to establish normal contractile behaviour in the bud. The degree of muscle process reorientation in a particular cell depends on its position within the bud. When cells of the parental polyp enter the bud on the upper or lower side (facing the head or foot of the mother polyp), their muscle processes are properly oriented in both parent and bud. Muscle processes of cells entering the bud at lateral positions have to reorient by up to 90° with respect to their future orientation in the bud (Otto, 1977).

**Fig. 2.**
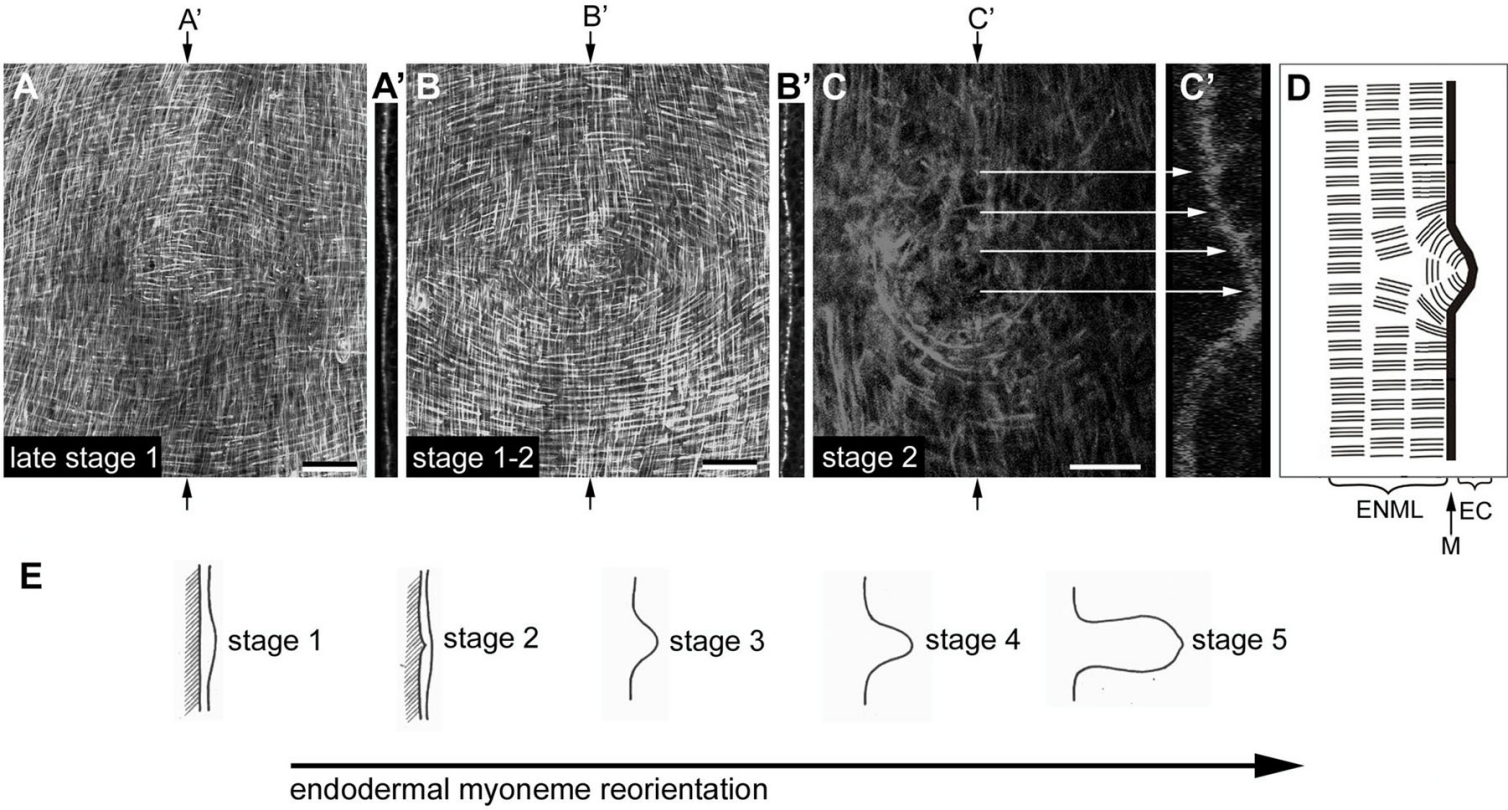
**Early circular arrangement of endodermal muscle processes.** (A,B,C) Confocal projections of the basal regions of ectodermal and endodermal epithelial cells of the budding zone stained with Alexa Fluor 488 Phalloidin. (A) Late stage 1 bud, in which ectodermal muscle processes run vertically and endodermal ones run horizontally. In the centre, endodermal muscle processes start to deviate from their regular orientation and appear shortened. (B) Eye-shaped arrangement of endodermal muscle processes in bud stage 1-2. (C) Circular orientation of endodermal muscle processes in bud stage 2, which reflects the correct endodermal planar polarity within the newly forming polyp. (A′,B′,C′) Orthogonal optical sections of (A,B,C) at the positions indicated by arrows show that endodermal bending into the ectodermal layer is correlated with circular arrangement of endodermal muscle processes. (D) Scheme of the arrangement of endodermal muscle processes at bud stage 2. ENML, endodermal muscle layer; M, mesoglea; EC, ectodermal layer. (E) Timing of endodermal muscle process reorientation according to bud stages from Otto and Campbell (1977). Depth of confocal projections: 20 µm in A; 15 µm in B; 25 µm in C. Scale bars: 50 µm in A and B; 20 µm in C.

Bud initiation starts with a thickening of both epithelial layers in a circular area in the lower body column (Fig. S4A-D). Endodermal muscle processes appeared more condensed in the centre of the thickened area in late stage1 buds, and they started to deviate from the regular arrangement by forming an eye-shaped array placed in the centre (Fig. 2A,B). These changes occurred before the basal muscle layers associated with the mesoglea exhibited any visible curvature (Fig. 2A′,B′). Shortly after, in stage 2 buds, endodermal muscle processes established a circular arrangement (Fig. 2C), which coincided with an endodermal bending towards the ectodermal layer (Fig. 2C′,D). Ectodermal epithelial cells lying directly over these endodermal cells shortened their apical-basal diameter (Fig. S5A-C) and, as a result, the outer surface of the tissue bilayer did not show obvious curvature. From stage 3 on, evagination proceeded in both epithelial layers to form a macroscopically visible bud protrusion (Fig. S5D).

**Fig. 3.**
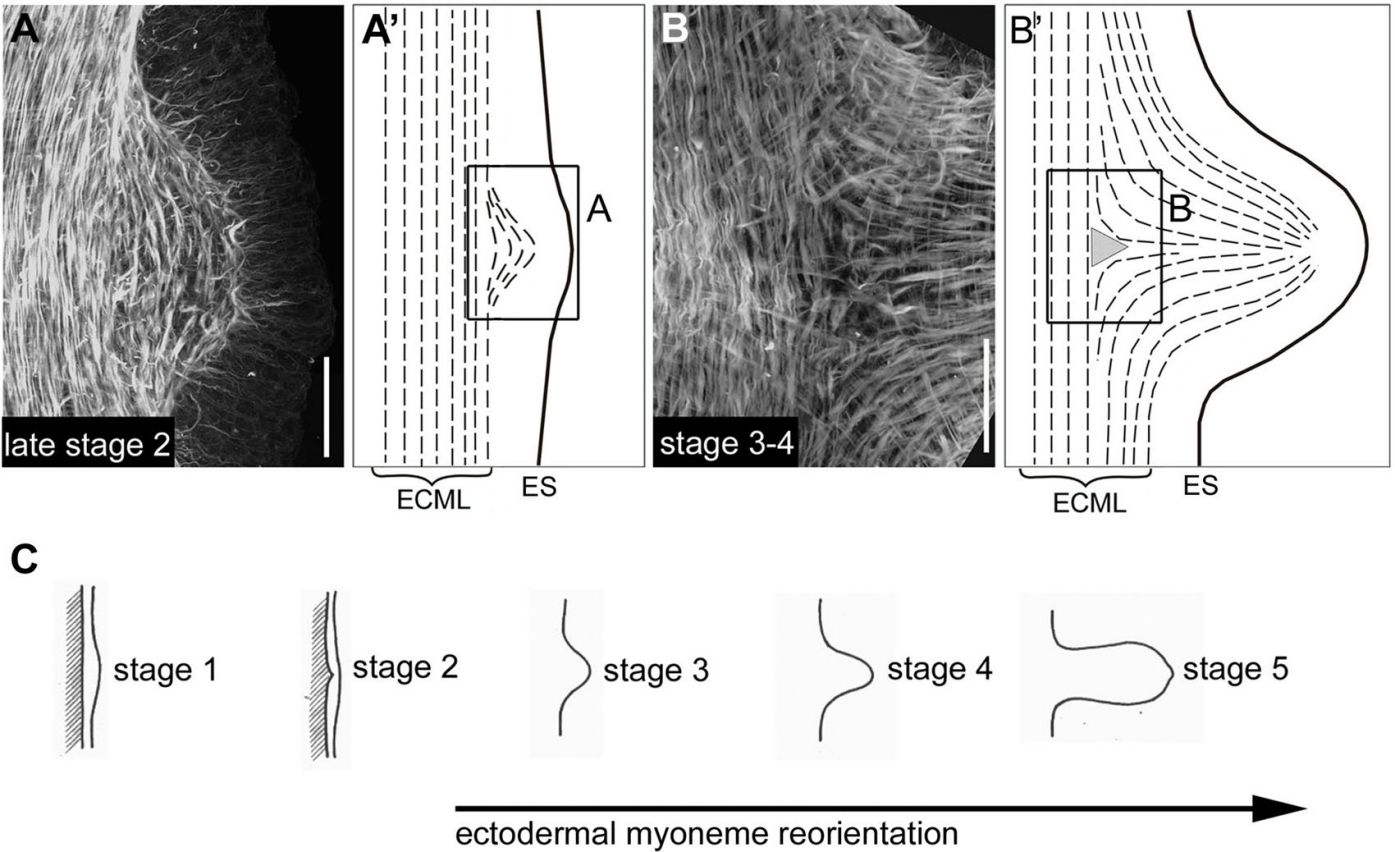
**Lateral views of early bud stages showing ectodermal muscle process reorientation.** (A,B) Confocal projections of specimens stained with Alexa Fluor 488 Phalloidin. (A′,B′) Schematic drawings of ectodermal muscle processes (dashed lines) at the corresponding stages. While ectodermal muscle processes moving into the bud from oral and aboral directions are oriented rather correctly with respect to the buds new body axes, those entering the bud from lateral areas have to increasingly rearrange in order to attain proper orientation. This results in a triangle shape at bud stage 3-4. (C) Timing of ectodermal muscle process reorientation according to bud stages from Otto and Campbell (1977). Projection depths: 30 µm in A; 10 µm in B. ECML, ectodermal muscle layer; ES, ectodermal apical surface. Scale bars: 50 µm.

Ectodermal muscle processes maintained their original orientation until bud stage 2, when they were still aligned as in the adjacent parental tissue. The distance between them, however, increased as a result of the curvature of the developing protrusion (Fig. 3A) (Otto, 1977). Starting at bud stage 3, ectodermal muscle processes began to reorient their polarity until they established a bud-specific axial alignment (Fig. 3B). A triangular area was visible at the base of stage 3 and older buds, where muscle processes aligned parallel to the parental axis were separated from those aligning with the newly formed axis of the bud (Fig. 3B,B′) (Otto, 1977).

### Reorientation of muscle processes during tissue recruitment

**Fig. 4.**
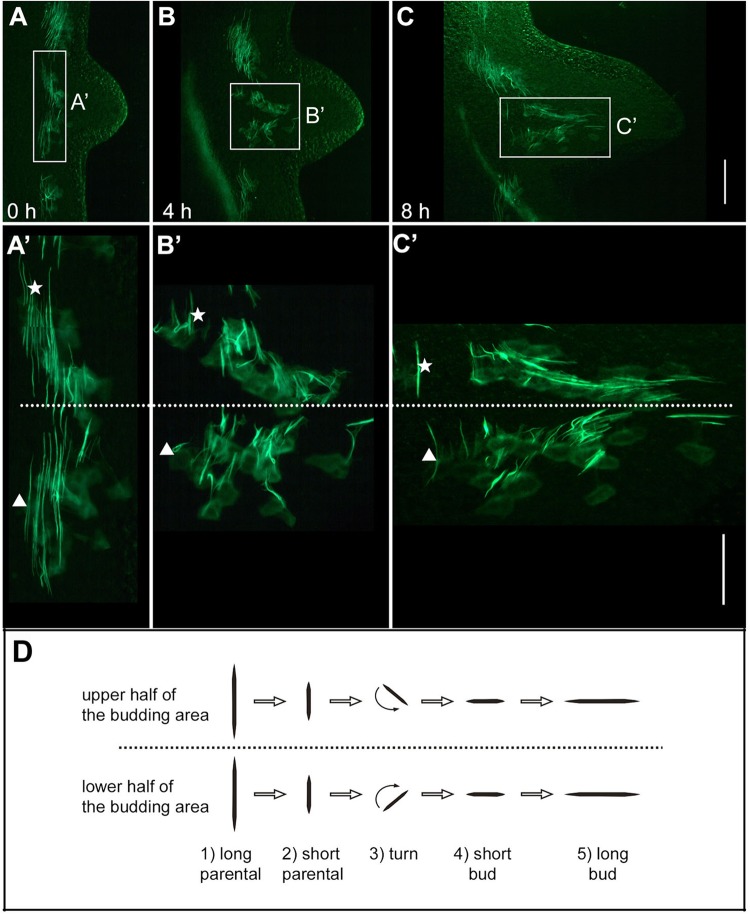
**Reorientation of ectodermal muscle processes during bud evagination.** (A,B,C) Live imaging of a developing bud at three time points (0, 4, 8 h) showing muscle processes in patches of transgenic cells. (A′,B′,C′) Magnified images reveal the rotational direction and contraction during reorientation in the upper (oriented towards the head of the mother polyp) and lower (oriented towards the foot of the mother polyp) half of the bud. Stars and triangles label two individual muscle processes in order to demonstrate converging movement. (D) Schematic summary of the events. Scale bars: 200 µm in A, B and C; 100 µm in A′, B′ and C′.

The fast outgrowth of the bud between stages 3 and 6 is due to recruitment of parental tissue by roughly concentric rings of epithelial cells moving towards the centre of evagination (Otto and Campbell, 1977). Due to this movement pattern, individual epithelial cells have to rearrange relative to each other by lateral intercalation (Otto and Campbell, 1977; Philipp et al., 2009). In order to track the behaviour of actin structures during lateral intercalation, we followed changes in muscle process orientation *in vivo* using Lifeact-GFP transgenic animals. Ectodermal muscle processes moving into the bud laterally reoriented in a distinct spatial pattern (Fig. 4). Cells located in the lower side of the evaginating bud (oriented towards the parental foot) turned their muscle processes clockwise; cells in the upper side (oriented towards the parental head) turned them counter-clockwise (Fig. 4A′,B′,C′,D). In addition to a change in direction, muscle processes exhibited a transient reduction in length upon reorientation: they shortened during reorientation and elongated to original length after reorientation (Fig. 4A′,B′,C′,D).

**Fig. 5.**
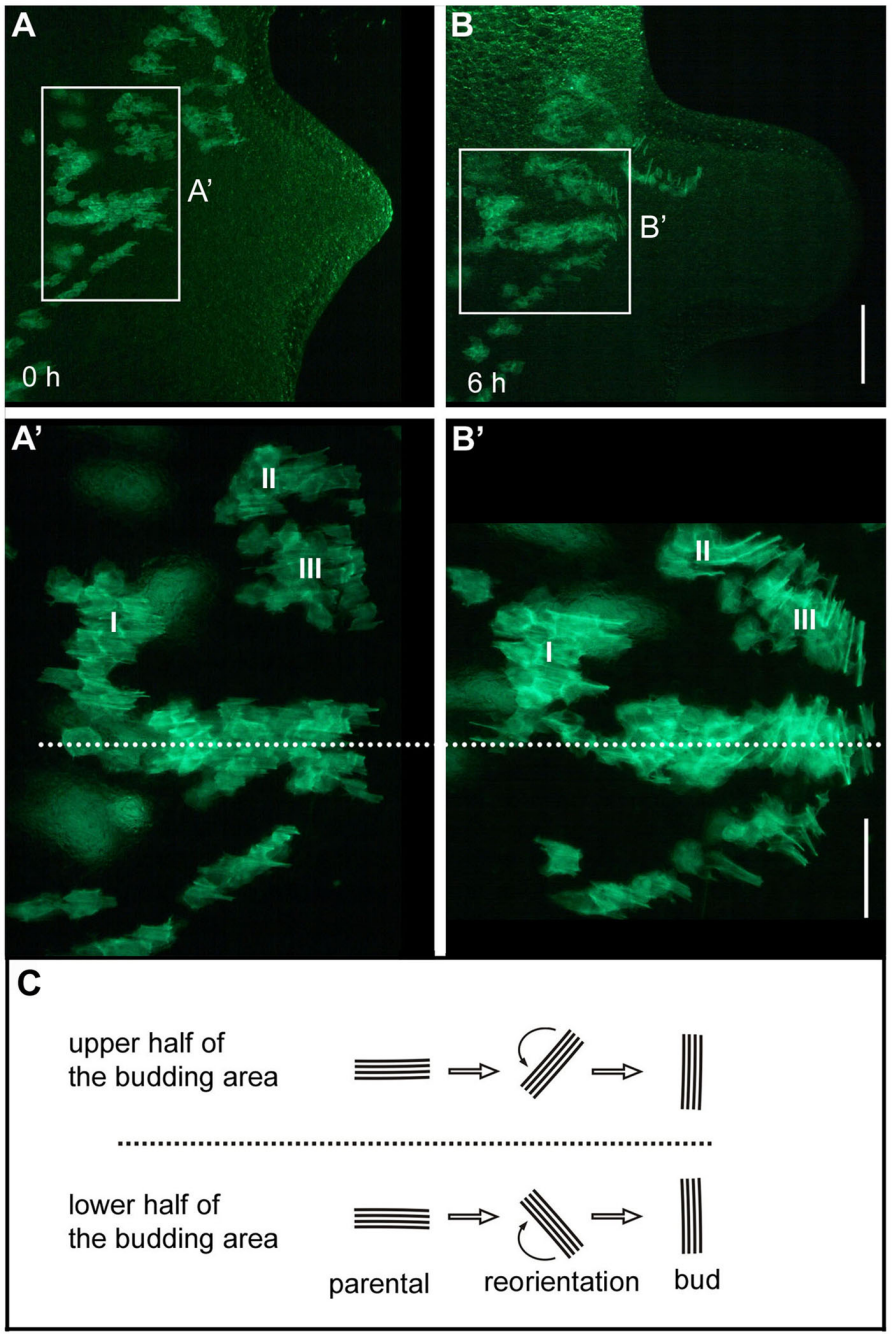
**Reorientation of endodermal muscle processes during bud evagination.** (A,B) Live imaging of a developing bud at two time points (0, 6 h) showing muscle processes in patches of transgenic cells. (A′,B′) Magnified images reveal the rotational direction during reorientation in the upper (oriented towards the head of the mother polyp) and lower (oriented towards the foot of the mother polyp) half of the bud. (C) Schematic summary of the events. Scale bars: 200 µm in A and B; 100 µm in A′ and B′.

Endodermal muscle processes showed basically the same behaviour (Fig. 5). Cells located on the lower side of an evaginating bud turned them clockwise, cells of the upper side counter-clockwise (Fig. 5A′,B′,C). Changes in muscle process length, however, were not observed in endodermal cells.

### Lateral intercalation is accompanied by polarized junctional remodelling

**Fig. 6.**
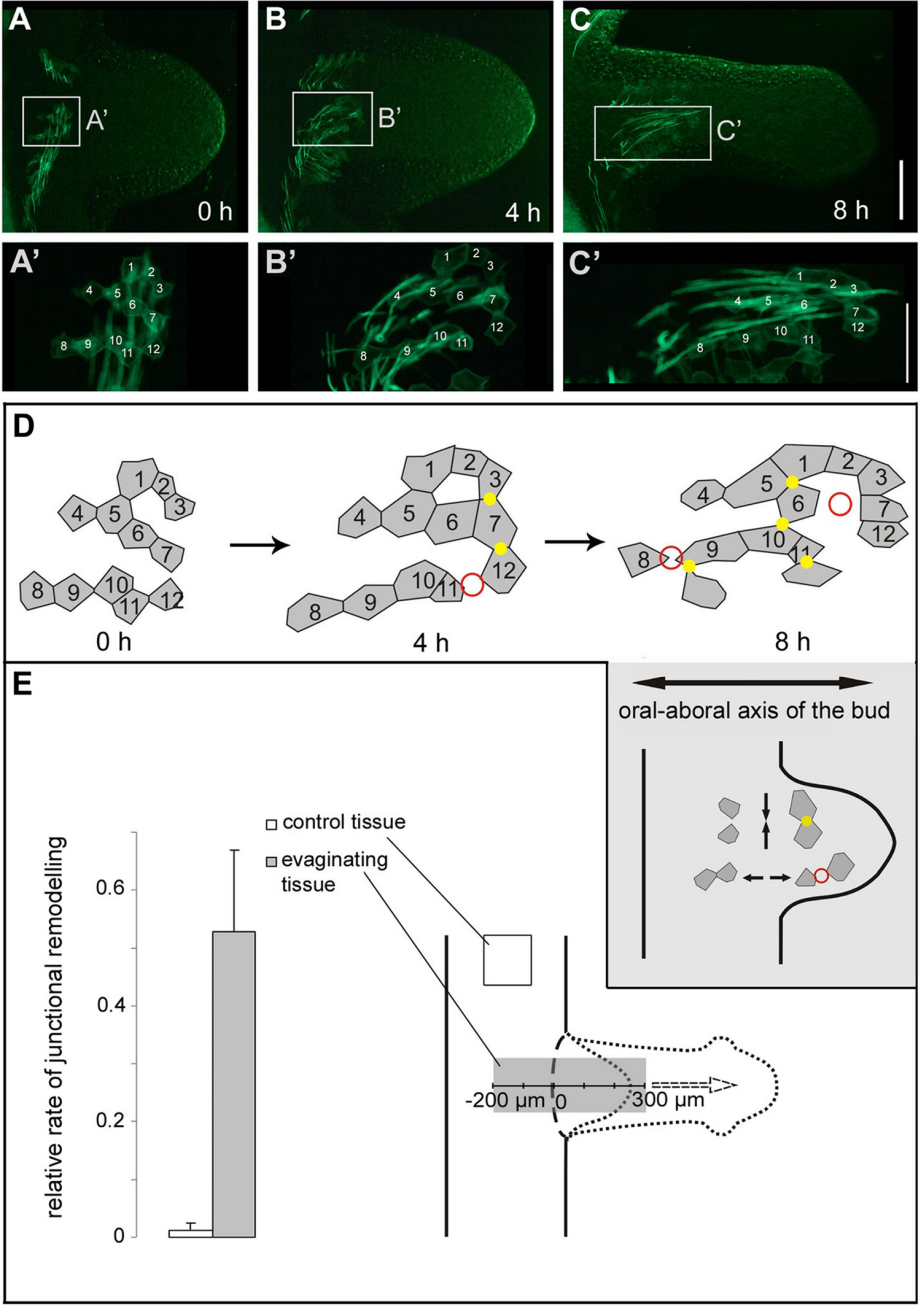
**Polarized remodelling of ectodermal apical cell junctions during bud evagination.** (A,B,C) Live imaging of a developing bud at three time points (0, 4, 8 h) showing a larger patch of transgenic cells. (A′,B′,C′) Magnified images are focused on F-actin associated with apical septate junctions; individual transgenic cells are labelled with numbers. (D) Schematic drawings of the transgenic cells in A′, B′ and C′, highlighting their positions and contact areas. Yellow dots mark new cell-cell contacts, which are established along the newly developing oral-aboral axis of the bud. Red circles mark detachment of existing cell-cell contacts, oriented perpendicular to the oral-aboral axis of the bud. (E) Relative rate of apical junctional remodelling in the evaginating area (grey bar) as compared with control tissue in the mid-gastric region (white bar). To determine the rate of remodelling, the number of polarized changes (yellow dots + red circles) per existing cell-cell contact between two transgenic ectodermal epithelial cells was counted during a period of 12 h. For evaginating cells, a total of six cell clusters including 110 cell-cell contacts were analyzed. During the tracking period, these cells moved from roughly −200 µm distance to the bud-parent border within the mother polyp to roughly 300 µm distance to the bud-parent border in the bud, and about one out of two cells either connected to a new cell neighbour or lost contact to its previous neighbour. For control cells, a total of three cell clusters including 157 cell-cell contacts were analyzed. These cells showed much slower movement along the oral-aboral axis of the mother polyp, and changed their neighbours at a very low rate. Scale bars: 200 µm in A, B and C; 100 µm in A′, B′ and C′.

We also studied junctional remodelling in ectodermal epithelial cells by tracking the relative positions of the apical actin belts connected to septate junctions (Fig. 6). During lateral intercalation, apical contacts were established between formerly unconnected cells along the bud's new oral-aboral axis (Fig. 6A′,B′,C′,D, yellow dots). Furthermore, formerly connected cells detached in an orientation perpendicular to this axis (Fig. 6A′,B′,C′,D, red circles; Movie 3). This intercalation behaviour resulted in an extension of the tissue along the bud's axis (Fig. 6A′,B′,C′). Junctional remodelling occurred in a large area covering the parental tissue and the evaginating bud, but was not observed outside of the budding zone (Fig. 6E).

As in the ectoderm, recruitment of endodermal tissue into the bud requires rearrangement of epithelial cells relative to each other. Here, microscopic resolution was limited and our observations deep in the *Hydra* endodermal tissue layer could not resolve changes in apical actin belts at the single cell level. Nevertheless, we did observe clear changes in Lifeact-GFP-positive endodermal cell clusters. As in the ectoderm, they narrowed along the axis of the parental polyp and extended along the newly formed bud axis due to lateral intercalation of adjacent cells (Fig. 5).

### Ectopic ectodermal muscle processes are associated with cell motility

**Fig. 7.**
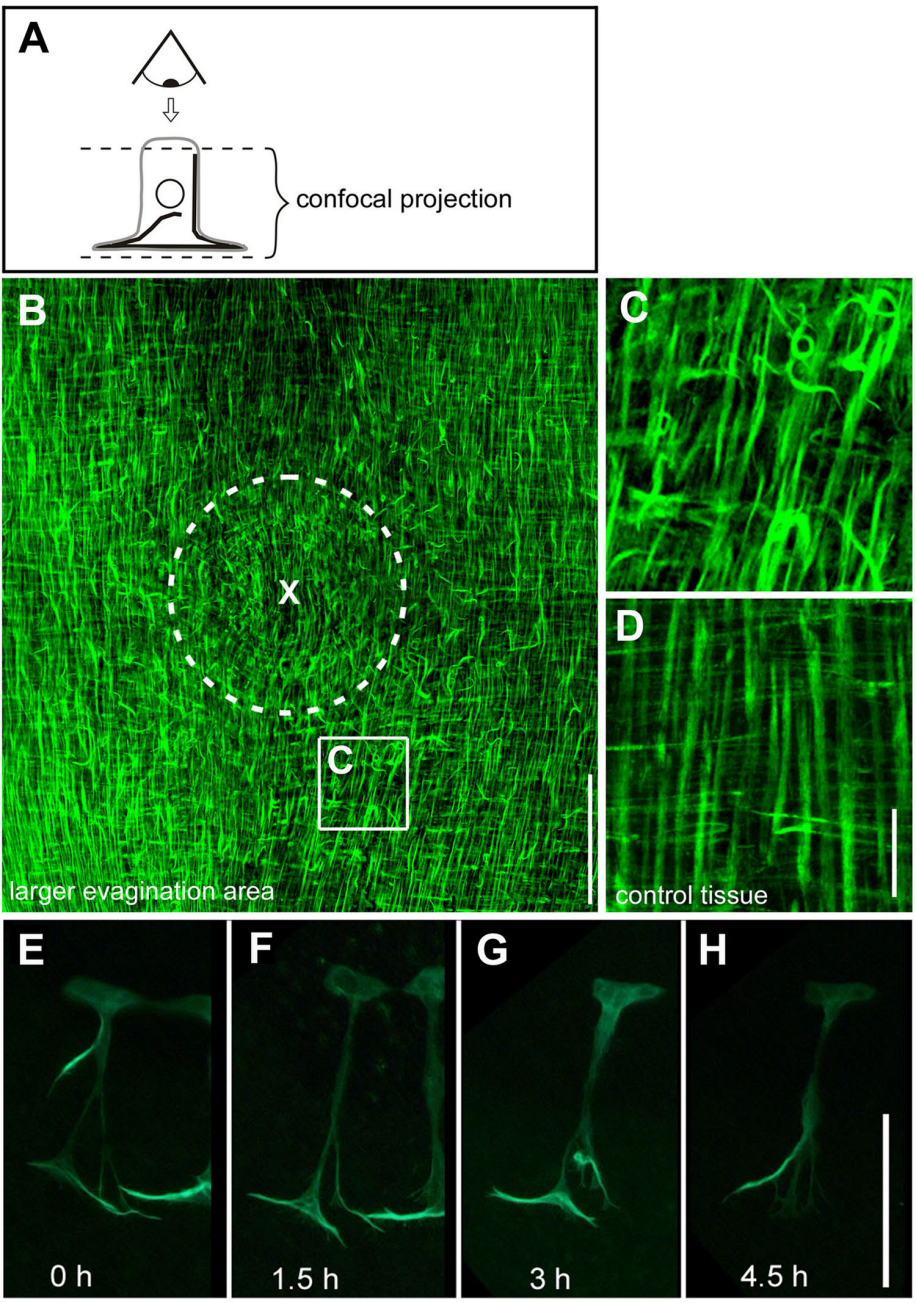
**Randomly oriented ectodermal muscle processes in the larger budding area.** (A) Schematic showing the perspective of the observer on the plane of the epithelium along an outside-inside direction. Confocal projection for B-D starts directly below the apical septate junctions of ectodermal epithelial cells and extends towards the basal end of endodermal epithelial cells including their muscle layer. Samples were stained with Alexa Fluor 488 Phalloidin. (B) Confocal projection of a stage 2-3 bud and the surrounding tissue showing a large number of muscle processes bent or randomly oriented. X marks the centre of evagination; the dotted circle indicates the bud-parent border. (C) Magnified view as indicated in B. (D) Regular arrangement of muscle processes in unevaginated control tissue from the mid-gastric region. (E-H) Short interval live tracking of an individual ectodermal epithelial cell during tissue recruitment shows dynamic remodelling of its actin fibers. Scale bars: 100 µm in B; 20 µm in C and D; 100 µm in E-H.

During tissue recruitment, individual ectodermal muscle processes not only reoriented, but displayed highly irregular polarity and spatial arrangements (Fig. 7). These detached fibres were found in evaginating bud tissue, but also at positions outside of the evaginating tissue in the region between bud and parental polyp (Fig. 7A-C). They were not present in morphogenetically inactive regions of the polyp (Fig. 7D). High magnification live tracking of individual ectodermal cells revealed that the subcellular localization of detached muscle processes was dynamically remodelled in time intervals of a few hours supporting a view that they contribute to cell movement (Fig. 7E-H). In addition, they extended in an apical-basal direction up to the apical actin belt (Fig. 7E). Therefore they may also be involved in coordinating apical and basal parts of a moving epithelial cell.

## DISCUSSION

Dynamics of actomyosin networks underlie many morphogenetic cell behaviours (Guillot and Lecuit, 2013; Heisenberg and Bellaiche, 2013; Munjal and Lecuit, 2014). Therefore, considerable effort has been made to develop methods for live imaging of F-actin. In order to specifically label F-actin networks at highest resolution, we produced stable transgenic *Hydra* polyps expressing Lifeact*-*GFP in ectodermal and endodermal epithelial cell lineages. Our approach follows previous attempts to create whole animal Lifeact models to study organismic physiology and development (Riedl et al., 2010; Phng et al., 2013). We have cultivated these Lifeact-GFP strains for several years and have not observed any significant impairment of polyp behaviour, reproduction, or regeneration. The mosaic Lifeact-GFP polyps used in this study produce buds with the same staging pattern and frequency as wild-type animals. We found identical F-actin dynamics at specific budding stages in Lifeact-GFP animals and phalloidin-stained wild-type preparations, indicating that the presence of Lifeact-GFP did not noticeably affect the behaviour of actin filaments. Bud formation, however, occurred at a slightly lower rate in fully transgenic polyps. We observed that these polyps catch and eat smaller numbers of brine shrimp per day as compared to wild-type polyps. This could be due to a marginal interference of Lifeact-GFP with actin contractility involved in nematocyte discharge or movement of tentacles.

### Epithelial F-actin architecture in transgenic Lifeact-GFP *Hydra*

Ectodermal and endodermal epithelial cells are surrounded by an apical F-actin belt associated with septate junctions. Septate junctions perform two major functions in *Hydra*: restricting trans-cellular permeability and mediating intercellular adhesion (Wood, 1985). Linking F-actin to septate junctions is likely to provide stability against mechanical forces and to connect actin networks between neighbouring cells in order to transmit force at the apical plane of the epithelium. In ectodermal epithelial cells, we detected a fine network of smaller actin filaments just below the apical surface connecting the F-actin belt across the entire planar cell diameter. The function of this network has not yet been explored, but an involvement in the control of apical cell shape seems likely. In addition, we found dynamic actin-filled filopodia extending outward from the apical F-actin belt, which may engage in the formation and re-organization of cell contact sites between neighbouring cells.

By enhancing the resolution of earlier studies, which had used maceration preparation and ultrathin serial sectioning (Mueller, 1950; David, 1973; West, 1978), Lifeact-GFP-expressing ectodermal epithelial cells show two to three long, 1-2 µm thick muscle processes. Endodermal epithelial cells, in contrast, exhibit a higher number of shorter and much finer muscle processes covering the entire basal surface. At the ultrastructural level, ectodermal and endodermal muscle processes have been demonstrated to be similarly constructed, containing filaments with the same diameter (Haynes et al., 1968). Thus, a different degree of actin fibre condensation between ectoderm and endoderm is probably based on differential use of scaffold and linker proteins.

### Actin dynamics during tissue bending

*Hydra* bud formation can be separated into three phases with presumably phase-specific morphogenetic processes: initiation, elongation, and detachment. Gierer (1977) argued that a 2D cell sheet such as the gastric body wall of *Hydra* is in a state of biomechanical stability, so that a distinct mechanical force (‘bending moment’) is required to break the flat state and to cause curvature. von Gelei (1925) was the first to provide a detailed histological description of the earliest bud stages. He described thickening of the ecto- and endoderm in the prospective budding area to form a flat, placode-like structure, which was later confirmed by Webster and Hamilton (1972) and Graf and Gierer (1980). In the planula larva of the anthozoan *Nematostella vectensis*, Fritz et al. (2013) recently showed that tentacle evagination also starts in a placode-like structure of thickened ectodermal epithelial cells. von Gelei (1925) further described a distinct behaviour in a group of 10-20 endodermal epithelial cells in the centre of the thickened area, which substantially decreased their apical-basal diameter and started to bend towards the ectodermal layer. Based on this, he postulated that endodermal pressure towards the ectoderm provided the initial mechanical force for bending. Campbell (1967) speculated that these cell shape changes in the endoderm result from a decrease in lateral affinity between the cells and an increase in affinity for the extracellular matrix, which would create a bending moment in the endoderm towards the ectoderm. Notably, Sherrard et al. (2010) found that apical-basal shortening of endodermal cells drives invagination during *Ciona* gastrulation, and in this case the observed cell shape changes were clearly caused by actomyosin contractility.

**Fig. 8.**
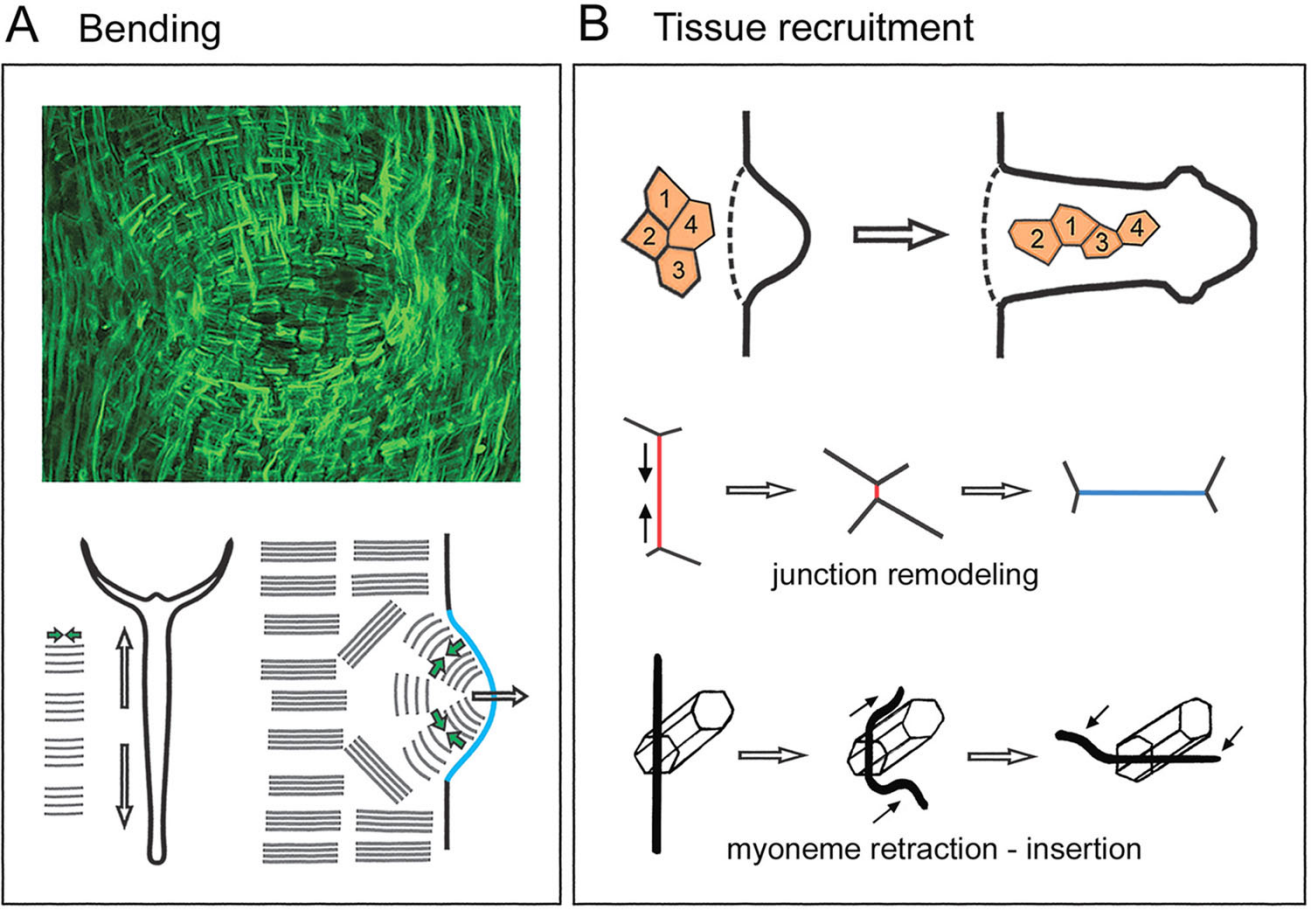
**Actin-based processes involved in bud morphogenesis.** (A) Bending. Circular arrangement and contractile activity of basal muscle processes in a group of endodermal epithelial cells may participate in initiating tissue bending. The blue line in the lower scheme represents the mesoglea, and the ectodermal layer is not shown. The confocal image on a stage 2 bud is taken from Philipp et al. (2009) with permission from PNAS. (B) Tissue recruitment. Movement of epithelial cells towards the centre of evagination during bud elongation and tissue recruitment involves apical junction remodelling and retraction, re-polarization and re-attachment onto the mesoglea of basal muscle processes.

In addition to apical-basal shape changes, Otto (1977) conjectured that contraction of ring-shaped endodermal muscle processes may contribute to a displacement of the endoderm towards the ectoderm, analogous to the elongation of the body column by contraction of endodermal muscle processes. The data presented here and in our previous study (Philipp et al., 2009) agree surprisingly well with this model. Contraction of an endodermal muscle process ring formed by bud stage 2 could promote the initial bending of the endodermal layer (Fig. 8A). Notably, endodermal muscle process rings are also formed, when tentacles start to evaginate in developing buds and during head regeneration (Fig. S4E-G), and when ectopic tentacles start to appear along the body column in alsterpaullone-treated polyps (Philipp et al., 2009; Anton-Erxleben et al., 2009).

What are the molecular signals underlying these changes in cellular behaviour? Thickening in both layers of the bud placode and apical-basal shape changes in endodermal cells are graded responses with strongest expression of the phenotype in the centre of the placode (Fig. S4A-D; von Gelei, 1925; Philipp et al., 2009). This centre will later develop into the evaginating tip, and we propose that it may act as a point source for instructive signals. In a preceding study (Philipp et al., 2009), we suggested that secreted Wnt5 proteins may induce changes in shape and polarity in adjacent endodermal cells. *wnt5* is expressed in a small group of ectodermal epithelial cells located directly above the responding endodermal cells, and *wnt5* activation is directly correlated with the induction of tissue evagination during bud and tentacle formation. Furthermore, Wnt/PCP signalling is a major source of polarity cues throughout bilaterian developmental models (Keller, 2005; Zallen, 2007; Gros et al., 2009). Notably, transcriptional activation of *wnt5* occurs in ectodermal epithelial cells just prior to the first morphological signs of tissue bending, while changes in cell shape and cell polarity are first observed in the endodermal layer. Such clear functional partitioning along the outside-inside direction of the epithelial bilayer ensures correct bending (Gierer, 1977). In fact, bud invagination has never been observed in *Hydra.* Bending of a bud always occurs in the correct outward direction, whereas single-layered systems such as the sea urchin gastrula appear to be more error-prone and can sometimes bend in the wrong direction and hence produce exogastrula phenotypes (Gustafson and Wolpert, 1963).

### Actin dynamics during tissue recruitment

Elongation of the early bud is driven by recruitment of epithelial tissue from the mother polyp into the newly forming protrusion (Fig. 8B). Epithelial cells exhibit lateral intercalation and move in a convergent extension-like manner along a roughly circular fate map: cells located near the evaginating centre will end up in the oral/distal part of the bud; those located more distantly will move to a more aboral/proximal part of the bud (Otto and Campbell, 1977). The mechanisms generating this movement are unclear. During steady-state tissue turnover, epithelial cells in the mid-gastric region are displaced together with the underlying mesoglea along the oral-aboral axis towards the foot (Aufschnaiter et al., 2011). With the initiation of a bud, however, cells in the budding zone change their direction and start to move from all sides towards the future centre of evagination. The mechanisms generating this movement are unclear. Campbell (1980) proposed a hypothesis that epithelial cells actively crawl on the mesoglea by using their contractile muscle processes. Recent support for this view came from observations of epithelial cells moving into the bud relative to the underlying mesoglea (Aufschnaiter et al., 2011). Possible ‘molecular clutch’ mechanisms of integrin-based force transmission to an extracellular matrix have been reviewed recently (Case and Waterman, 2015). Here, we show that indeed a large fraction of ectodermal muscle processes in the budding area is detached from the mesoglea and disconnected from neighbouring cells. They are present at any radial position in the bud ruling out that they represent axially reorienting muscle processes. Furthermore, they remodel within short time intervals. An engagement of such detached muscle processes in cell motility is further supported by their presence in evaginating tentacles and during head regeneration, two other processes involving cell migration (R.A. and B.H., unpublished data).

Lateral cell intercalation has been thoroughly investigated in embryonic epithelia of various bilaterians with a particular focus on apical (Drosophila) and lateral (vertebrates) actin dynamics (Munjal and Lecuit, 2014). The data presented here, however, provide evidence that a force for morphogenetic movement may be generated in the basal part of the epithelium by contractile muscle processes. Similar phenomena have actually been described in rare cases, during epithelial cell rearrangement in the dorsal hypodermis of *Caenorhabditis elegans* (Williams-Masson et al., 1998), during convergent extension of the notochord in ascidians (Munro and Odell, 2002), and during convergent extension of the mouse neural plate (Williams et al., 2014). In all these examples, epithelial cells form actin-based protrusions at a basolateral position, and this basal protrusive activity clearly acts in intercalation movements. Despite obvious structural differences between basolateral, filopodia-like protrusions and basal muscle processes, they emphasize a potentially underestimated and more general role of actin dynamics in the basal compartment of epithelial and epitheliomuscular cells for tissue morphogenesis.

We tried to interfere with cytoskeletal dynamics by using a set of actin and myosin inhibitors including CytochalasinD, Latrunculin, Swinholide and Blebbistatin. However, when polyps with a stage 1-2 bud were treated with inhibitor, none of them implemented specific inhibition of bud initiation or tissue recruitment. It was difficult to assess the effect of the myosin inhibitor Blebbistatin, because it totally blocked contractile behaviour in the polyp and halted any tissue movement including budding and early head regeneration (Fig. S6A). Among the actin inhibitors, CytochalasinD strongly blocked wound healing during early head regeneration (Fig. S6B), but had no obvious effect on bud evagination in the regular concentration range of up to 25 µM. We interpret this result as an inability of CytochalasinD to interfere with existing muscle processes and apical actin belts in the evaginating bud tissue.

The epithelial movement pattern observed during tissue recruitment clearly needs a directional cue, which is undefined at present, but which we presume is an attractive signal located at the evaginating centre. Candidates for such a signal, which are defined by their local expression in the evaginating centre of a young bud and by their known role in establishing directional cues in bilaterian development, include Wnt, FGF and Nodal signalling factors (Hobmayer et al., 2000; Lengfeld et al., 2009; Philipp et al., 2009; Lange et al., 2014; Watanabe et al., 2014). During tissue recruitment, ectodermal and endodermal muscle processes finally reinsert and elongate in proper orientation with respect to the bud's oral-aboral axis in order to re-establish proper contractility of the body column of the new animal. In a parallel set of experiments, we have studied *de novo* formation of muscle processes in *Hydra* reaggregates. The results show that the establishment of parallel actin fibers at the basal surface of epithelial cells is initially a cell-autonomous event. Thereafter, coordinated alignment of muscle processes within larger fields of cells follows the formation of a new oral-aboral polyp body axis (Seybold et al., 2016; Livshits et al., 2017). Hence, the head organizer as primary signalling centre for axial patterning may be responsible for providing this directional cue in *Hydra* aggregates and could do so during bud development.

Finally, cells changing their positions relative to each other by lateral intercalation need to continuously modify their apical junctional complexes in order to maintain the stability, integrity and permeability of a cellular sheet. We observed epithelial junctional remodelling during tissue recruitment, and its spatial dynamics corresponds to the pattern described in bilaterian tissues (Fig. 8B). It should be noted that bilaterian junctional remodelling has been studied in great detail in cadherin-based adherens junctions (Takeichi, 2014). In *Hydra*, however, these apical complexes are represented by septate junctions, the structure of which is currently not precisely known. Preliminary data emphasize co-localization of beta-catenin and other members of the cadherin-catenin-adhesion complex at *Hydra* septate junctions (Broun et al., 2005; S. Pontasch and B.H., unpublished data), but also reveal the presence of claudin (M.-K. Eder and B.H., unpublished data). Due to these differences in structure and composition, apical remodelling of septate and adherence junctions must use different molecular mechanisms.

### Conclusion

In- and evagination of tissues are fundamental mechanisms to generate shape in metazoan embryos and during organogenesis. The underlying actin behaviour has been analyzed in great detail in embryos of various bilaterian model organisms, and mechanisms such as apical constriction and lateral intercalation have been shown to cause tissue bending and tissue movements. In this report, we have studied actin dynamics during bud evagination in the body wall of the simple metazoan *Hydra*. Asexual reproduction using ‘adult’ tissue is a common feature among ancestral animal lineages. Hence, our findings may add to a general understanding of shape-generating mechanisms in animal development. The body wall of *Hydra* differs from bilaterian embryonic tissue layers in two major aspects: it is built as an epithelial double layer and the individual unit is a differentiated epitheliomuscular cell with at least two contractile actin structures, an apical adhesion belt and basal muscle processes. Based on the ability of both structures to create contractile forces not only within a single cell but over larger groups of cells, changes in tissue shape can be generated in the apical and basal compartments of the two epithelial layers. We propose coordinated action of two processes. First, a group of endodermal epithelial cells uses apical-basal shortening and a contractile muscle process ring to break the 2D stability of the double layer and to initiate tissue bending. Second, movement of laterally intercalating epithelial cells towards the centre of evagination forces the bud to protrude in an outward direction. Since it is obviously difficult to define distinct, F-actin-based forces simply by observation, it will be essential in a next step to improve functional interference with key molecular factors and to develop enhanced imaging tools.

## MATERIALS AND METHODS

### Hydra culture and sex induction

Wild-type *Hydra vulgaris* (strain Basel) and sex inducible *Hydra vulgaris* (female AEP and male PA-2 strains) (Martin et al., 1997) were propagated in asexual mass culture under daily feeding at a constant temperature of 18±1°C as described (Hobmayer et al., 1997). For sex induction, AEP polyps were transferred to a reduced feeding regime as described (Hobmayer et al., 2001). Egg and sperm production in the animals started approximately 2 weeks after initiation of the reduced feeding regime.

### Generation of stable transgenic Lifeact-GFP Hydra strains

A construct containing the actin-binding domain from yeast Abp140 ‘Lifeact’ (Riedl et al., 2008) fused in frame to GFP was injected into fertilized eggs of *Hydra vulgaris* (AEP) according to Wittlieb et al. (2006). In order to prepare the construct, the yeast peptide sequence MGVADLIKKFESISKEE plus a linker sequence GDP were translated into a DNA sequence taking into account the codon usage of *Hydra* (Fig. 1A; www.kazusa.or.jp/codon/). The resulting short DNA-fragment was amplified by PCR using long, overlapping primers and inserted into the GFP-expression vector ‘hotG’ (*Hydra actin1* promoter) (Wittlieb et al., 2006), in front of and in frame with the EGFP sequence. This construct was injected into fertilized *Hydra* eggs at the one- to two-cell stage. The injected eggs were kept at 18°C in hydra culture medium for 2-3 days until the cuticle stage was reached (Martin et al., 1997). Then, they were transferred to 12°C for 2 weeks and raised to 18°C for another week. Under these conditions, the animals started to hatch. Three injection series were performed. Overall, 74 eggs were injected with the Lifeact-GFP expression construct, and about half of the injected embryos hatched within a time period of 5 weeks after injection. Twelve of these hatchlings exhibited stable, mosaic expression of Lifeact-GFP in ectodermal or endodermal epithelial cells, interstitial cells or a combination of the three. The mosaic animals exhibited normal morphology, morphogenesis and regeneration, and were not distinguishable in behaviour from nontransgenic control animals. Asexual bud formation and selection of buds enriched in transgenic cells were used to raise polyps completely transgenic in the ectodermal and endodermal cell lines. We also injected the hotG expression construct with untagged GFP as a control. In epithelial cells of these transgenes, localization of untagged GFP showed uniform distribution throughout the cytoplasm (data not shown).

### Phalloidin labelling

Animals were relaxed in 2% Urethane in hydra culture medium, fixed for 1-2 h at 4°C with 4% PFA in culture-medium, washed 3×10 min in PBS, followed by a treatment with 0.1% Triton X-100 in PBS (PBS-T). Whole mount staining or staining of manually isolated budding regions using Alexa Fluor 488 Phalloidin and Rhodamine Phalloidin (Molecular Probes, Eugene, USA) diluted 1:100 or 1:200 in PBS-T, was carried out for 1 h at room temperature. After three washes in PBS, the specimens were mounted in Vectashield antifade mounting medium (Vector Laboratories, Burlingame, USA).

### *In situ* hybridization

Visualization of the spatial expression patterns of *Hydra actin1* and *actin2* genes by *in situ* hybridization was performed as described (Philipp et al., 2005).

### Microscopy

Living transgenic animals and fixed specimens were observed under an LSM 510 confocal microscope (Zeiss, Oberkochen, Germany) or an MZ 16F stereomicroscope (Leica, Wetzlar, Germany). TIRF images and movies were captured on an iMIC-stand (Till Photonics, Gräfelfing, Germany) with a 100×/1.45 NA objective (Olympus, Tokyo, Japan) using a 300 mW Argon laser. Prior to microscopy, living transgenic animals were anesthetized in 2% urethane in culture medium for 2-5 min, mounted on a microscopic slide and lightly squeezed with a cover slip to bring the tissue into a flattened focal plane. After a maximum of 10-15 min of live imaging, the animals were allowed to recover in fresh culture medium. When immobilized on microscope slides beyond this time span, the epithelial bilayer started to show deteriorating integrity and loss or damaging of epithelial cells. During tracking experiments, animals were therefore repeatedly anesthetized and mounted for each individual tracking time point.
